# Presentation and Management of Acute Mania in Fanconi–Bickel Syndrome, A Metabolic Genetic Disorder

**DOI:** 10.1155/2024/5593846

**Published:** 2024-04-04

**Authors:** Allen P. F. Chen, Geoffrey Russell, Amnie Ashour, Adeeb Yacoub

**Affiliations:** ^1^Medical Scientist Training Program, Renaissance School of Medicine, Stony Brook, NY, USA; ^2^Department of Psychiatry, Renaissance School of Medicine, Stony Brook, NY, USA; ^3^The Division of General Surgery at New York-Presbyterian, Brooklyn, NY, USA

## Abstract

Fanconi–Bickel syndrome (FBS) is a rare metabolic disorder caused by decreased glucose transporter 2 (GLUT2) function due to several known mutations in the *SLC2A2* gene. As of 2020, 144 cases of FBS have been described in the literature. Metabolic and somatic sequelae include dysglycemia and accumulation of glycogen in the kidney and liver. However, there are no descriptions in the literature of possible neuropsychiatric manifestations of FBS. This case report is to our knowledge the first in this regard, describing a patient with FBS who was admitted to our psychiatric inpatient unit while experiencing acute mania. We conceptualize the case as a novel psychiatric presentation of acute mania in FBS, which may inform our understanding of bipolar disorder pathophysiology because of the hypothesized functional changes in neural pathways involving the paraventricular thalamus induced by decreased GLUT2 activity in FBS.

## 1. Introduction

Fanconi–Bickel syndrome (FBS) is a rare autosomal recessive metabolic disorder caused by mutational loss of function of glucose transporter 2 (GLUT2), which is highly expressed in the gut but also detectable and functionally important in other areas, including certain brain regions. The classic presentation of FBS is characterized by dysglycemia and its various sequelae, but there is no literature on neuropsychiatric manifestations of FBS. Here, we describe to our knowledge, the first report on the identification and treatment of acute mania in an FBS patient. We further describe this presentation and the neurobiological ramifications underlying the FBS metabolic perturbation.

Metabolic disturbances have long been hypothesized to be linked to psychiatric disorders. Supporting this, metabolic syndromes related to blood glucose dysregulation are more prevalent in people with psychiatric disorders [1–3], but direct links between metabolic pathways and mood remain unclear. Recent research has uncovered the vast roles of glucose transporters in brain function and disease [4]. Dysfunction of GLUT2 is implicated in Type II diabetes mellitus (T2DM) [5]. There is a bidirectional epidemiological association between T2DM and bipolar disorders [6, 7]. There is also an increased risk of mood and psychotic disorders in patients with T1DM [8, 9]. Furthermore, autoantibodies to GLUT2 may exist in the early course of type 1 diabetes, but it is probably a transient epiphenomenon with no clear connection to neuropsychiatric symptoms [9–11]. Pathophysiological mechanisms relating specifically GLUT2 function to mood or cognitive disorders remain elusive. Part of this knowledge gap is tied to a lack of tractable genetic models and metabolic-targeted interventions for mood disorders [12–14]. Consistent with this, there has been a lack of behavioral studies specifically investigating the impact of genetic-metabolic manipulations on behavior.

This case report describes a patient with FBS who was admitted to our psychiatric inpatient unit on two separate occasions with presentations resembling acute mania typically seen in bipolar I disorder. The patient's psychiatric presentation may be explained by neuronal signaling changes caused by the loss-of-function mutation in the GLUT2 gene underlying FBS because of the presumptive role of GLUT2 in the regulation of mood neural networks involving the paraventricular thalamus, amygdala, and nucleus accumbens. Combining experimental findings from mouse models and nonhuman primates with the likely involvement of the nucleus accumbens and amygdala in the brain pathophysiology of bipolar disorders, we hypothesize that mania is a novel neuropsychiatric presentation of FBS.

## 2. Case Presentation

Mr. C is a 28-year-old male of consanguineous parents who was diagnosed with FBS as a child and presented to our psychiatric emergency department with severe acute mania. Being a relatively rare genetic condition, to our knowledge, this report is the first to describe the identification and treatment of psychiatric symptoms in FBS. He has resulting chronic kidney disease, cardiac complications, hepatomegaly, postprandial hyperglycemia, and fasting hypoglycemia requiring frequent meals. At the time of the psychiatric evaluations at our hospital, he was living at home with his parents and younger brother and had been married once but was divorced. He attended college for a time and then went on to work with his father in the family construction business. There is no family history of mood disorders or other mental illness.

Mr. C began demonstrating impulsivity and aggression in early adolescence. The first psychiatric hospitalization was at the age of 21, when he experienced a severe major depressive episode. He has since been hospitalized numerous times due to recurrent manic episodes characterized by severe irritability, impulsivity, and violent behavior. The patient had a history of sporadic cocaine, benzodiazepine, and cannabis use, but he was not using any substances at the time of the hospitalizations.

Mr. C was admitted to our psychiatric unit twice. The first admission was preceded by an episode during which the patient unprovokedly and repeatedly punched his father in the head and then chased his father while threatening to kill him. In the psychiatric emergency room, he attacked other patients and staff repeatedly and without provocation. He was treated emergently with haloperidol, lorazepam, fluphenazine, chlorpromazine, and diphenhydramine throughout the emergency department course. The mental status examination on admission demonstrated severe irritability, grandiosity, recurrent violent behaviors, psychomotor agitation, racing thoughts, and paranoia. Treatment was started with fluphenazine 10 mg twice daily and valproic acid 500 mg in the morning and 1,000 mg at night (valproic acid serum level was 99). Fluphenazine decanoate was also administered. The patient returned to euthymia after 10 days, and he was discharged.

Six days after discharge, the patient again presented to the psychiatric emergency room. According to his father, he was not taking the fluphenazine and valproic acid after discharge. He subsequently stopped sleeping and returned to a similar manic state characterized by severe irritability, grandiosity, impulsivity, psychomotor agitation, violent behavior, and paranoia. While in the psychiatric emergency room, an episode was described during which the patient appeared calm, asked a nurse a question, and then began wildly punching the nurse and other staff without explanation. During this second inpatient admission, he was restarted on fluphenazine 10 mg twice daily and valproic acid 500 mg in the morning and 1,000 mg at night. The patient returned to euthymia after 12 days and was discharged.

## 3. Discussion

**Figure 1:**
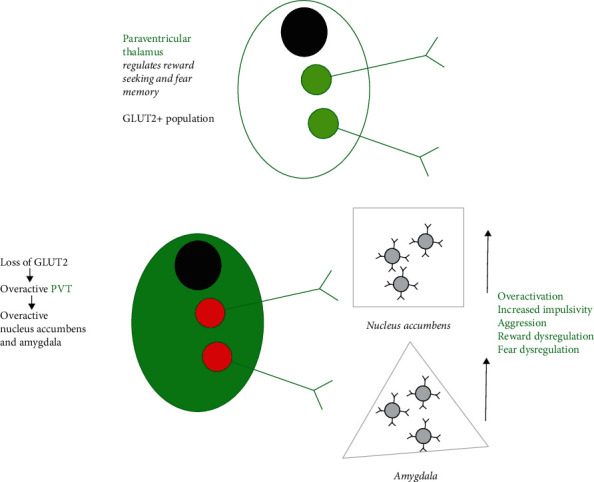
A loss of GLUT2 induces and overactivation of the PVT-to-nucleus accumbens and PVT-to-amygdala circuit, contributing to acute mania and impulsivity in FBS. As demonstrated by Labouebe et al. [17], the PVT harbors a GLUT2+ glutamatergic population. Critically, they demonstrated that GLUT2 inactivation or local hypoglycemia induces activation of the PVT. On top is a cartoon depiction of the PVT, which has been found to regulate reward seeking and fear memory. The PVT neurons facilitate these behaviors by innervating regions involved in such as the nucleus accumbens and amygdala. On the bottom, we depict what may occur in FBS, with the chronic loss of GLUT2 inducing an overactive PVT network. Thus, we propose that FBS patients may exhibit a chronically overactive PVT and thus dysregulate regions involved in motivational salience and emotional processing such as the amygdala and nucleus accumbens. An overactive amygdala/nucleus accumbens circuit may thus contribute to manic episodes.

First described by Fanconi and Bickel, glycogen Storage Disease XI, more commonly known as FBS, is a rare glucose metabolism disorder caused by several known loss-of-function mutations in *SLC2A2*, the glucose transporter 2 (GLUT2) gene [15, 16]. FBS is rare, having been identified in less than 200 patients worldwide. This case report describes a patient with FBS who was admitted to our psychiatric hospital with manic episodes resembling bipolar I disorder. We explore how the FBS mutation and glucose dysregulation may lead to bipolar disorder. Considering human, nonhuman primate, and rodent model studies, we hypothesize that the loss of function of GLUT2 in FBS may explain this novel neuropsychiatric presentation of acute mania and impulsivity; based on these studies, loss of GLUT2 function may lead to resultant overactivity of the paraventricular thalamus (PVT) and downstream brain structures and thus impulsivity and manic manifestations (Figure 1).

In understanding how FBS may lead to bipolar manifestations such as acute mania and impulsivity, the PVT is a major brain region that expresses GLUT2. Furthermore, the PVT is an emerging center for reward-seeking, and a portion of its function is under regulatory control by metabolic signaling, specifically via GLUT2. Rodent model studies have revealed that the PVT contains a major GLUT2-expressing, glucose-sensing neuronal population [17]. Inactivation of GLUT2 in this neuronal population leads to overactivation of the PVT and a downstream structure, the nucleus accumbens. It was first found that genetic inactivation of GLUT2 in neurons enhanced sucrose-seeking behavior in mice, which led to the discovery of GLUT2-expressing PVT neuron projections to the nucleus accumbens, a brain region strongly linked to reward and motivation. The firing rates of PVT GLUT2+ neurons increase in response to states of hypoglycemia. These neurons were subsequently characterized as glutamatergic, activating the nucleus accumbens when firing in response to hypoglycemia. Activation of the nucleus accumbens in turn facilitates a reward-seeking and thus high-motivation state. These data suggest a feedback mechanism that enforces sucrose-seeking behavior in nutrient-deprived states by way of PVT-to-nucleus accumbens signaling. This feedback loop was under the control of GLUT2, such that GLUT2 inactivation led to compulsive reward seeking.

A recent study further demonstrated that upregulated anterior PVT activity increases compulsive sucrose seeking in mice [18]. The authors treated mice with a high-fat diet, which has previously been linked to neurometabolic changes that induce behavioral compulsive caloric consumption. Even in the presence of threatening and harming cues, these mice are biased toward a hedonic state in which they seek sucrose. In this high-fat diet-fed state, the PVT exhibited heightened activity, and inhibiting PVT activity resulted in attenuation of compulsive sucrose-seeking behavior [18]. Overall, these data reinforce the role of the PVT as a neural metabolic-sensing center that regulates reward-seeking behavior.

PVT connectivity and function have since been studied in further detail in nonhuman animals. The PVT is a nucleus in the dorsal midline thalamus serving as a node in limbic networks. Present understanding of the PVT includes important roles in the regulation of wakefulness and circadian rhythm, reward-related behavior, anxiety-like behavior, depression-like behavior, stress response, and arousal to novel stimuli [19]. Synaptic connections terminating on the PVT utilize several neurotransmitters classically associated with psychiatric disorders, including serotonin, norepinephrine, and dopamine. In its function as a node in the limbic network, the PVT integrates signals from a variety of emotionally charged stimuli and then provides excitatory inputs to brain regions involved in both positive and negative emotional states, such as the amygdala, nucleus accumbens, and prefrontal cortex, making it critical for emotional processing [19, 20]. Thus, the PVT has a supported role in impulsivity and possible neuropsychiatric manifestations of bipolar disorder.

The PVT has been characterized on human neuroimaging as a relatively small part of the thalamus, and only recently has it been identified as a distinct thalamic structure [21, 22]. Like the nonhuman PVT, these studies have also demonstrated that the human PVT has connectivity with the ventral striatum, prefrontal cortex, nucleus accumbens, and amygdala [21]. PVT activity has also been correlated consistently with states of behavioral arousal in humans [23]. The PVT may also serve as a node in the brain anxiety network, implying a potential role in defensive and combative behaviors [20].

Human neuroimaging studies have implicated overactivation of the thalamic network, nucleus accumbens, and amygdala in bipolar disorder. Overactivity of the amygdala has been identified consistently across bipolar disorder patients during acute mood episodes, interepisodically, and in response to emotional cues [24]. Activation of these regions has been shown to trigger impulsivity and reward-seeking behaviors, as well, which are important to the clinical descriptions of mania [25, 26]. Several prior neuroimaging studies support the notion that dysfunction of the nucleus accumbens may underlie impulsivity seen in several psychiatric conditions, such as bipolar disorders, borderline personality disorder, substance use disorders, and ADHD [27–29]. Thus, there is much evidence connecting bipolar disorder with overactivation of brain structures now considered downstream of the PVT, namely the amygdala and nucleus accumbens.

Considering all this, we hypothesize that the loss of function of GLUT2 in FBS may lead to an overactive PVT network, providing aberrant excitatory input to the amygdala and nucleus accumbens as well as other structures. This supports the possibility of mania as a novel neuropsychiatric manifestation of FBS as seen in this case considering the limbic overactivation both at rest and during acute mood episodes in patients with bipolar disorder. Additionally, the patient's marked response to antimanic treatments could signify a hypothetical FBS-related neurobiological endophenotype in which PVT-mediated limbic overactivity relates mania with *SCLC2* haploinsufficiency. We note here, that there has been a lack of reports of mood disorders in FBS patients. There are multiple possibilities for this. There are less than 200 cases of FBS identified worldwide, rendering it unlikely to be able to detect an association between FBS and mood symptoms [16]. Along with this, mania and psychiatric disorders in FBS may go unrecognized because many of the cases are pediatric reports, focusing on the diagnosis and treatment of FBS prior to the average age of onset of many neuropsychiatric disorders. Lastly, bipolar disorder, and mood overall, is multifactorial with many hidden contributory protective and exacerbating factors [30, 31]. While we highlight a potential mechanism, we emphasize that we do not believe this is a sole cause, with many biological and psychosocial mechanisms at play.

## 4. Conclusion

This case report describes a patient with FBS who was admitted to our psychiatric inpatient unit for treatment of acute mania resembling that typically seen in bipolar I disorder. This is the first case report of a potential neuropsychiatric manifestation of FBS. The hypothesis of how mania can be explained by FBS is that overactivity of the PVT due to reduced GLUT2 function may lead to increased excitatory input to several brain regions known to be aberrantly activated during mania, especially the amygdala and nucleus accumbens. This unique case highlights a potential link between mood and metabolism, supporting a role for glucose processing in mood manifestations.

## Data Availability

Availability of data and materials is not applicable for this study.

## Abbreviations

FBS: Fanconi–Bickel syndrome
GLUT2: Glucose transporter 2
PVT: Paraventricular thalamus.
